# Comparative Influences of Fluid and Shell on Modeled Ejection Performance of a Piezoelectric Micro-Jet

**DOI:** 10.3390/mi8010021

**Published:** 2017-01-13

**Authors:** Kai Li, Jun-kao Liu, Wei-shan Chen, Lu Zhang

**Affiliations:** 1 State Key Laboratory of Robotics and System, Harbin Institute of Technology, Harbin 150001, China; 14b908004@hit.edu.cn or sdcxlikai@126.com (K.L.); cws@hit.edu.cn (W.-s.C.); 2 Aero Engine Corporation of China (AECC), Harbin Dongan Engine Corporation Limited, Harbin 150001, China; zhanglu916hit@163.com

**Keywords:** piezoelectric micro-jet, ejection performance, modelling and simulation

## Abstract

The piezoelectric micro-jet, which can achieve the drop-on-demand requirement, is based on ink-jet technology and small droplets can be ejected out by precise control. The droplets are driven out of the nozzle by the acoustic pressure waves which are generated by the piezoelectric vibrator. The propagation processes of the acoustic pressure waves are affected by the acoustic properties of the fluid and the shell material of the micro-jet, as well as the excitations and the structure sizes. The influences of the fluid density and acoustic velocity in the fluid on the nozzle pressure and support reaction force of the vibrator are analyzed in this paper. The effects of the shell material on the ejection performance are studied as well. In order to improve the ejection performance of the micro-jet, for ejecting a given fluid, the recommended methods of selecting the shell material and adjusting excitations are provided based on the results, and the influences of the factors on working frequencies are obtained as well.

## 1. Introduction

The working process of the piezoelectric micro-jet is based on the inverse piezoelectric effect and the core component is the piezoelectric vibrator. Acoustic pressure waves are generated in the fluid when pulse voltages are applied on the piezoelectric material, and then the droplets are ejected out of the nozzle by the pressure waves. The piezoelectric micro-jet technology, which can realize drop-on-demand, is widely used in electric and electronic fields [1,2], the bearing lubrication field [3], the manufacturing field [4,5,6,7], and the biomedical field [8,9], thanks to its advantages of fast response, good stability, simple structure, etc.

The influences of the pulse voltage parameters on the ejection performance of the micro-jet are widely studied by researchers based on the analysis of the hydrodynamic characteristics [10,11,12,13,14]. However, the ejection performance of the micro-jet is also affected by the acoustic properties of the fluid and the acoustic impedance of the shell. The propagation processes of the acoustic waves in the cavity are affected by the properties of the fluid, and the reflection/absorption processes of the acoustic waves, mainly at the interface between the fluid and shell, are related to the acoustic impedance of the shell [15].

The influences of the fluid acoustic properties and acoustic impedance of the shell on the ejection performance are analyzed in this paper. Based on the analysis results, the methods of shell material selection and excitation adjustment are given, when ejecting fluid with different properties, so as to ensure the ejection intensity of the micro-jet. In order to ensure the restraint stiffness of the vibrator, the reaction forces of the vibrator under different conditions are studied as well.

As for different applications, the designed structure of the piezoelectric micro-jet are different. The results shown in this paper are mainly used for the given common structure style shown in the text. For other structures, results can be obtained according to the proposed analysis simulation method.

## 2. Structure and Methods

**Figure 1:**
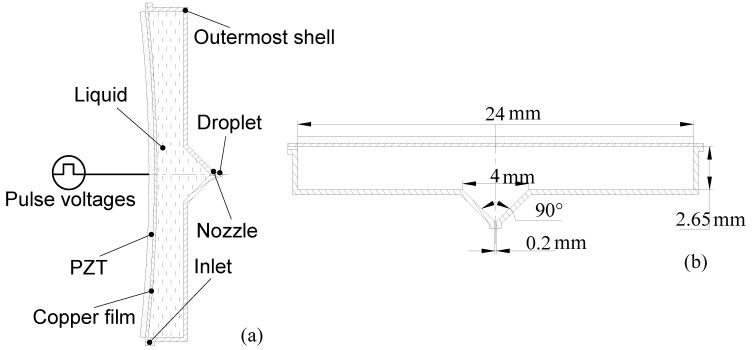
The structure and structure sizes of the piezoelectric: (**a**) the structure of the traditional piezoelectric micro-jet; and (**b**) the structure sizes of the micro-jet.

A piezoelectric micro-jet, which is simple and common, as shown in Figure 1a, is studied in this paper. The piezoelectric vibrator is made by gluing the piezoelectric ceramic (PZT) on the copper diaphragm. When pulse voltages are applied on the piezoelectric vibrator, the vibrator vibrates and acoustic pressure waves are created in the fluid domain. Then, the droplets are pushed out from the nozzle by the acoustic pressure waves. The structure sizes of the piezoelectric micro-jet are shown in Figure 1b. The thickness of the piezoelectric ceramic and copper diaphragm used in this paper are 0.4 mm and 0.2 mm, respectively.

**Figure 2:**
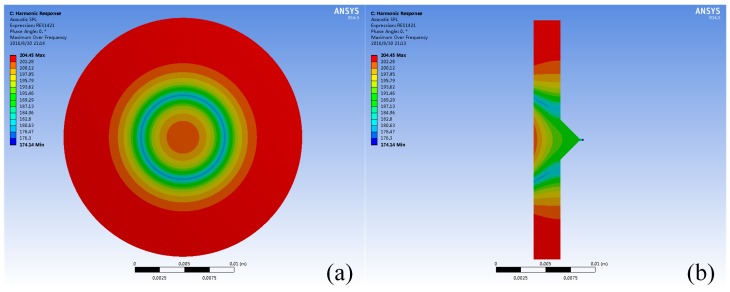
The acoustic pressure distribution in the fluid field of the piezoelectric micro-jet: (**a**) the global state of the pressure distribution; and (**b**) the cross-section diagram of the pressure distribution.

The acoustic pressure distribution in the fluid field of the piezoelectric micro-jet, after the vibration of the piezoelectric vibrator, is shown in Figure 2. As we can see, due to the transmission loss in the fluid domain and the reflection/absorption process at the interface between the fluid and shell, the acoustic pressure distribution in the cavity is not uniform, and the pressure level decreases gradually from the interface to the nozzle. As the pressure distribution is difficult to measure with sensors, it is difficult to study the acoustic pressure characteristics of the micro-jet through experiments. Thus, the correlation analyses are carried out by finite element analysis with simulation software ANSYS Workbench (Version 14.5, ANSYS, Inc., Canonsburg, PA, USA) which has been installed ExtAcoustics, ExtPiezo, and FSI_transient extensions.

## 3. Boundary Conditions

**Figure 3:**
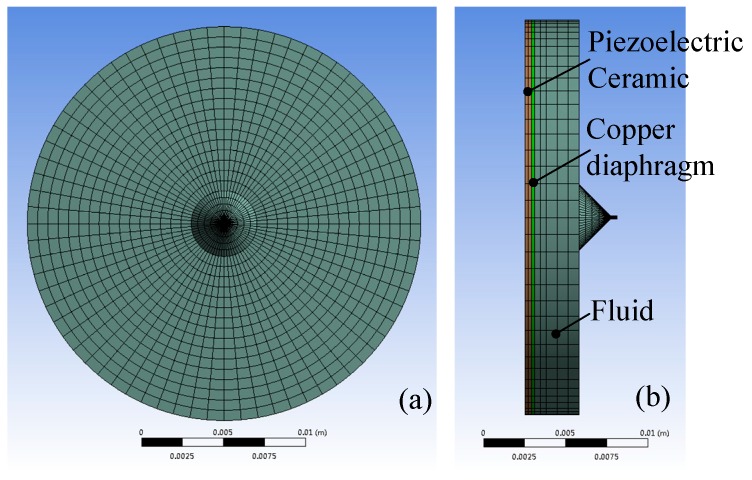
The model for simulation: (**a**) the front view of the model; (**b**) the left view of the model.

The model used in simulations is shown in Figure 3, we can see that the inlet part of the micro-jet is neglected due to its small size, and the sweep method is selected as the meshing method to improve the accuracy of calculations. As the effects of shell on acoustic pressure waves are determined by the acoustic impedance of the shell, the impedance boundary conditions are applied on the surfaces of the fluid domain instead of using the shell element.

The type of the piezoelectric ceramic is selected as PZT-5H, and its physical properties (elastic stiffness constant matrix [*c*^E^], piezoelectric stress constant matrix [*e*], and dielectric constant matrix [*ε*^T^]) in the simulation are set as:

$$\left[c^{E}]=\left[\begin{array}{cccccc} 13.2 & 7.3 & 7.1 & 0 & 0 & 0\\ 7.3 & 13.2 & 7.1 & 0 & 0 & 0\\ 7.1 & 7.1 & 11.5 & 0 & 0 & 0\\ 0 & 0 & 0 & 2.6 & 0 & 0\\ 0 & 0 & 0 & 0 & 2.6 & 0\\ 0 & 0 & 0 & 0 & 0 & 3 \end{array}\right]\times 10^{10}(N/m^{2}\right)$$

$$\left[e]=\left[\begin{array}{ccc} 0 & 0 & -2.4\\ 0 & 0 & -2.4\\ 0 & 0 & 17.3\\ 0 & 0 & 0\\ 0 & 12.95 & 0\\ 12.95 & 0 & 0 \end{array}\right](C/m^{2}\right)$$

$$\left[\varepsilon^{T}]=\left[\begin{array}{ccc} 804.6 & 0 & 0\\ 0 & 804.6 & 0\\ 0 & 0 & 659.7 \end{array}\right]\times 10^{-11}(F/m\right)$$

The elasticity modulus, density, and Poisson ratio of the copper film are set as 7.65 × 10^10^ N/m^2^, 7.5 × 10^3^ kg/m^3^, and 0.32, respectively. The outer ring of the copper diagram is set as a fixed constraint. The nozzle is the interface between fluid and air; therefore, the acoustic impedance at the nozzle is set as 400 kg·m^−2^·s^−1^ according to the related data [16]. In order to obtain the influences of the acoustic impedance of shell on the ejection performance of the micro-jet, the shell materials with different impedance are selected and the impedance values of different materials are shown in Table 1. The pulse voltages are applied to the surface of the piezoelectric ceramic and the amplitudes of the voltages are set as 200 V. As structure-acoustic coupling is created at the interface between the fluid domain and the piezoelectric vibrator, the boundary condition at the interface is set as a fluid-solid interface.

## 4. Results and Discussion

When the cavity of the micro-jet is filled with liquid, the vibration of the vibrator will be subjected to the reaction force of the fluid at the fluid-solid interface due to the fluid-solid coupling effect. The obvious influence of the fluid-solid coupling effect on the vibration of the vibrator is that the resonant frequencies of the vibrator are all reduced.

**Figure 4:**
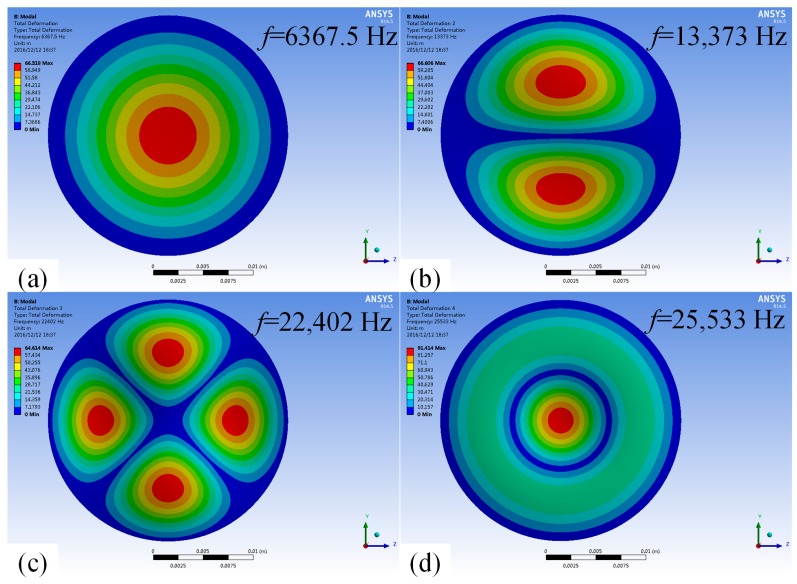
The mode shapes and the corresponding resonant frequencies of the vibrator when the cavity is filled with air: (**a**) first-order mode with the resonant frequency as 6367.5 Hz; (**b**) second-order mode with the resonant frequency as 13,373 Hz; (**c**) third-order mode with the resonant frequency at 22,402 Hz; and (**d**) fourth-order mode with resonant frequency at 25,533 Hz.

When the cavity is filled with air, the mode shapes and the corresponding resonant frequencies of the first four order modes of the vibrator are shown in Figure 4, we can see that in terms of the structure proposed in this paper, the first-order modal and the fourth-order mode are more suitable, as the nozzle of the piezoelectric micro-jet is located in the middle.

**Figure 5:**
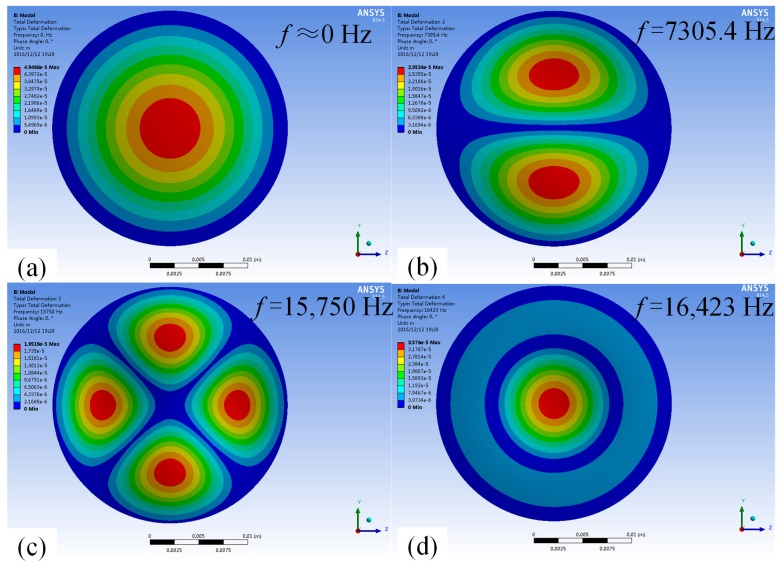
The mode shapes and the corresponding resonant frequencies of the vibrator when the cavity is filled with fluid: (**a**) first-order mode with the resonant frequency as close to 0 Hz; (**b**) second-order mode with the resonant frequency as 7305.4 Hz; (**c**) third-order mode with the resonant frequency as 15,750 Hz; and (**d**) fourth-order mode with the resonant frequency as 16,423 Hz.

When the cavity is filled with fluid, the mode shapes and the corresponding resonant frequencies are shown in Figure 5, and it can be seen that the resonant frequencies of the vibrator are all reduced significantly due to the fluid-solid coupling effect, while the mode shapes remain unchanged. The first-order working mode is suitable for low-frequency intermittent ejection, and the fourth-order mode is suitable for high-frequency continuous ejection.

As the constraint stiffness of the vibrator should meet the requirements, which are determined by the reaction force of the vibrator, so as to ensure the stability of the vibrator, the frequency response curves of the reaction force under different conditions are obtained. The frequency response characteristics of nozzle pressure are analyzed, as the ejection intensity is reflected by the acoustic pressure at the nozzle. To obtain better ejection performance, the vibration frequency of the vibrator, which determines the working frequency of the micro-jet, should be consistent with its resonance frequency. Thus, the influences of fluid properties and shell material on the resonant frequency of the vibrator are analyzed as well.

### 4.1. Influences of the Density of Fluid

**Figure 6:**
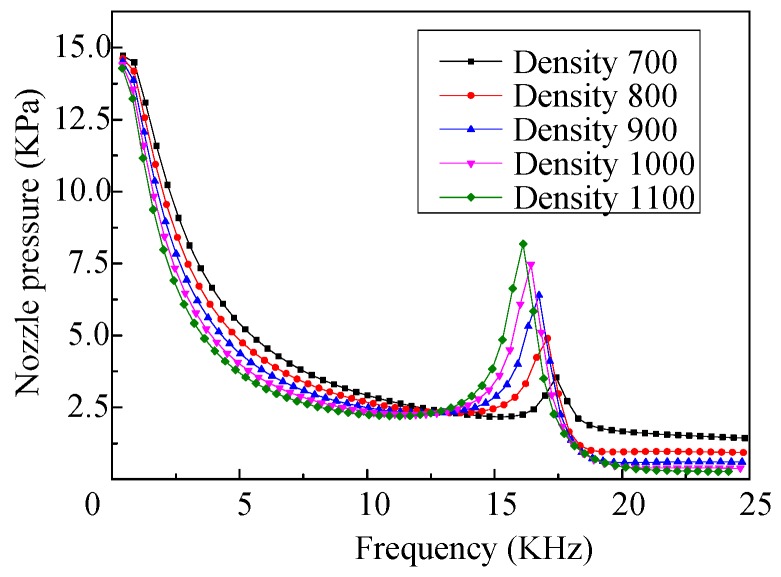
The frequency response curves of the nozzle pressure of the micro-jet (fluid with different densities).

The frequency response curves of the nozzle pressure of the micro-jet, when the acoustic velocity in the fluid is set as 1400 m/s and the material of the shell is set as aluminum, and when the fluids with different densities are ejected, are shown in Figure 6. As we can see that there are two peaks in each frequency response curve, which occur at the frequencies corresponding to the first-order and fourth-order resonant frequencies of the vibrator. It also demonstrates that the nozzle pressure of the micro-jet is less affected by the density of the fluids when the vibrator vibrates in the first-order mode.

**Figure 7:**
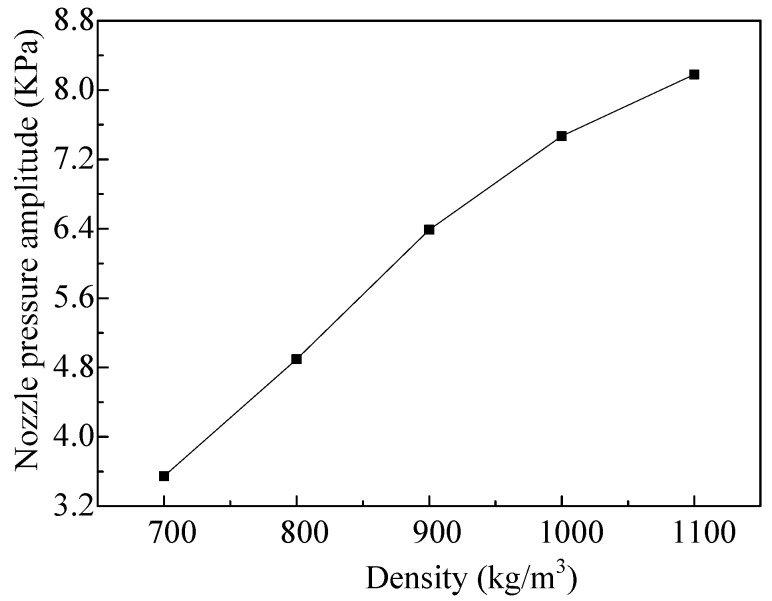
The amplitudes of the nozzle pressure vary with different densities of the fluid.

As the micro-jet generally operates at high frequency, so as to obtain high working efficiency, only the influences of the density on the nozzle pressure of the micro-jet, when the vibrator vibrates in the fourth-order mode, are analyzed. The effects of the densities on the amplitude of the nozzle pressure, when the vibrator vibrates in the fourth-order mode, are shown in Figure 7. We can see that with the increase of density, the amplitude of the nozzle pressure increases gradually, and the change trend is close to linear.

**Figure 8:**
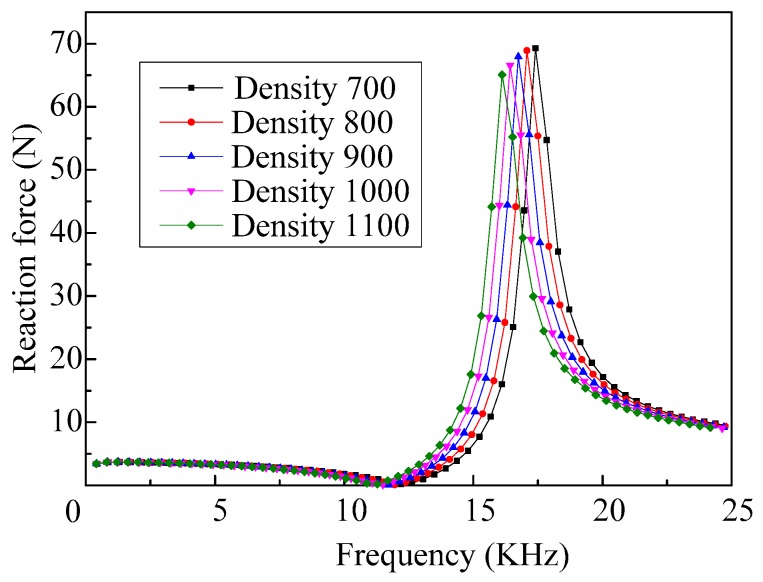
The frequency response curves of the reaction force of the vibrator when ejecting fluids with different densities.

The response curves of the reaction force of the vibrator, when fluids with different densities are ejected, are shown in Figure 8. We can see that there is only on peak value in each response curve of the reaction force of the vibrator and they all occur at the frequencies corresponding to the fourth-order mode. Additionally, the reaction force of the vibrator, when it vibrates in the first-order mode, is very small. The reason is that the inertia force created by the vibration of the vibrator, which determines the reaction force, relates to the vibration velocity of the vibrator, and the vibration velocity of the vibrator working in the fourth-order mode is larger than that of working in the first-order mode. Thus, the required restraint stiffness when the vibrator works in the fourth-order mode should be larger.

**Figure 9:**
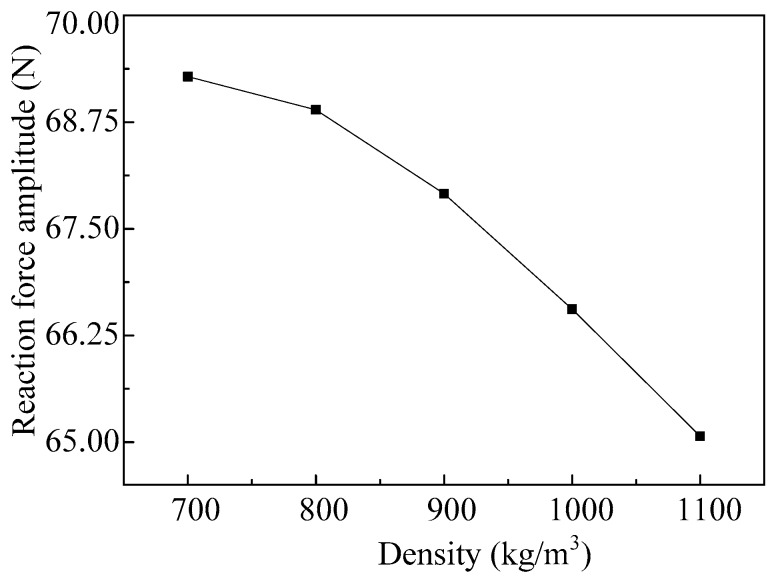
The amplitudes of the reaction force vary with different densities of the fluids.

As we can see from Figure 8, when the vibrator vibrates in the first-order mode, density has little effect on the reaction force of the vibrator. Thus, only the influences of the density, when the vibrator works in the fourth-order mode, on the ejection performance are analyzed here. The relationship between the reaction force amplitude of the vibrator which working in the fourth-order mode, and the densities of the fluids to be ejected is shown in Figure 9. As we can see, with the increase of the density, the amplitude of the reaction force decreases gradually, and the changes of amplitude increases.

**Figure 10:**
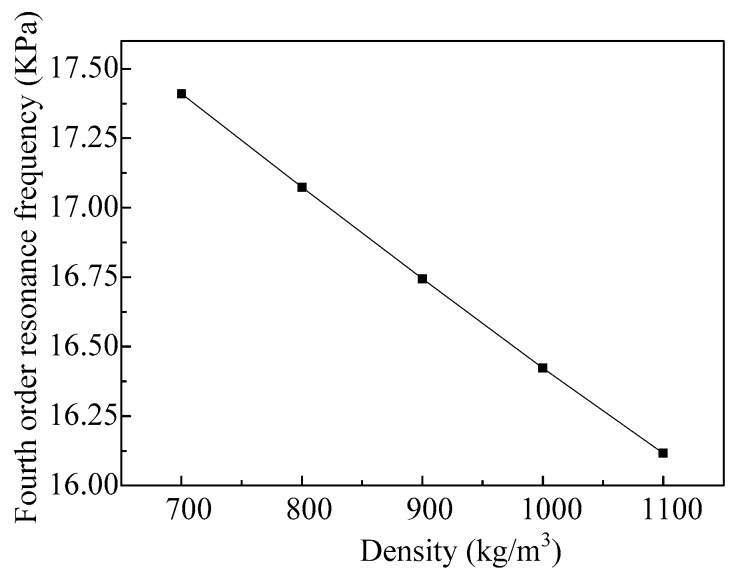
The fourth-order resonance frequencies of the vibrator varies with different fluid densities.

As we can see from Figure 6, when ejecting fluid with different densities, the resonant frequencies of the vibrator working in the first-order mode are different, but the difference is small, and the influences of the density on the resonant frequency of the vibrator working in the fourth-order mode are shown in Figure 10. We can see that the resonance frequency of the vibrator decreases nearly linearly with the increase of the density. Thus, the operating frequency of the micro-jet decreases when the density of the fluid increases.

### 4.2. Influences of the Acoustic Velocity in Fluid

**Figure 11:**
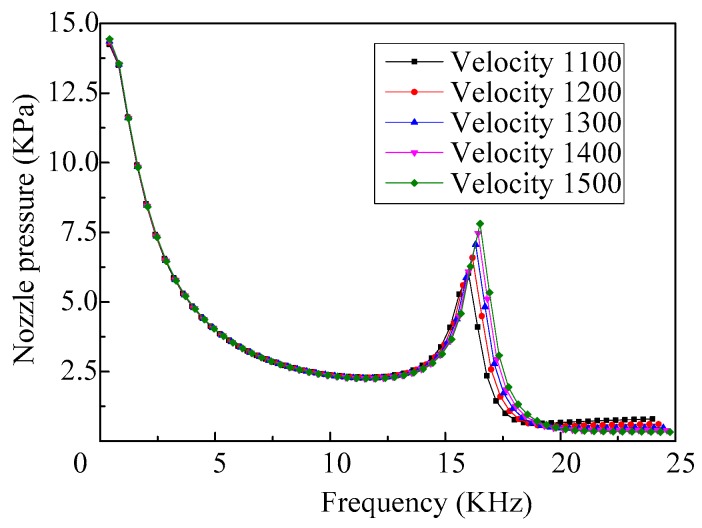
The frequency response curves of the nozzle pressure of the micro-jet (fluid with different acoustic velocity).

When the density of the fluid is set as 1000 kg/m^3^, the material of the shell is set as aluminum, and the acoustic velocity in the fluid is set differently, the frequency response curves of the nozzle pressure are shown in Figure 11. As we can see, there are also two peaks that occur in each frequency response curve, which correspond to the first-order and fourth-order modes. It can be seen that the frequency response curves of the nozzle pressure, when ejecting fluid with different acoustic velocity, is nearly the same. Therefore, when the micro-jet works at low frequency, the effect of acoustic velocity in the fluid on the nozzle pressure can be ignored.

**Figure 12:**
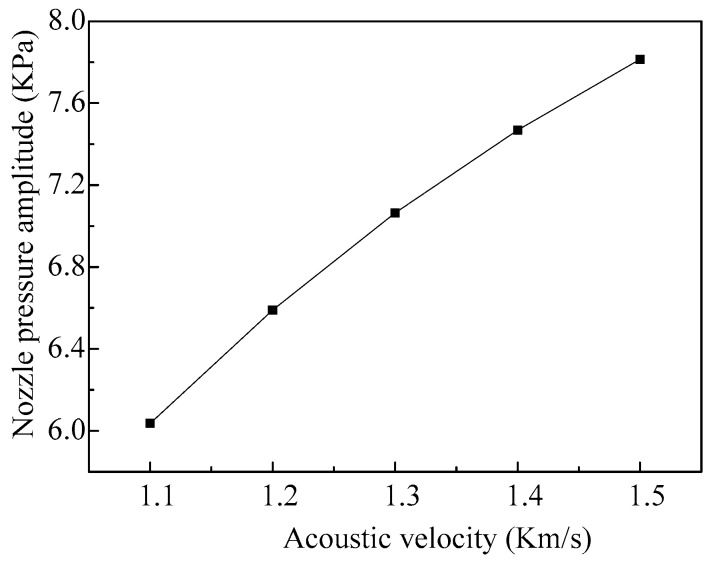
The amplitudes of the nozzle pressure vary with different acoustic velocity of the fluids.

As the acoustic velocity in the fluid has little influence on the nozzle pressure when the micro-jet is working at low frequency, only the relationships between the acoustic velocity and the nozzle pressure when the micro-jet is working at high frequency are obtained. The curve of the nozzle pressure amplitude along with the increase of the acoustic velocity of the fluid is shown in Figure 12. We can see that with the increase of the acoustic velocity, the amplitude of the nozzle pressure increases gradually and the change trend is close to linear.

**Figure 13:**
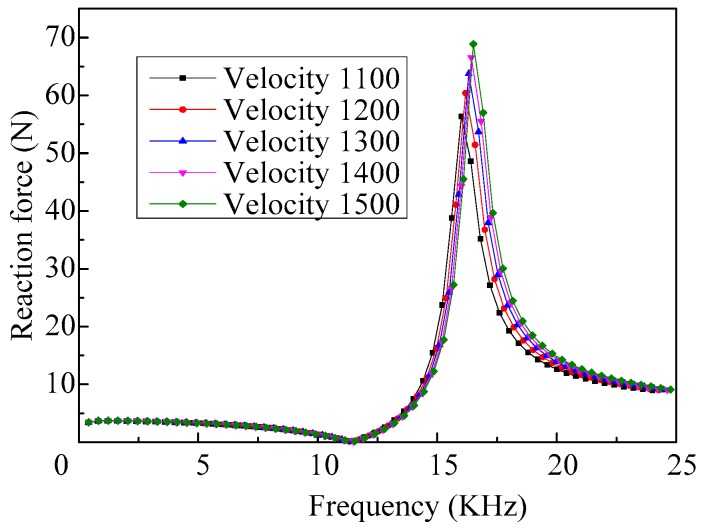
The frequency response curves of the reaction force of the vibrator when ejecting fluids with different acoustic velocities.

The response curves of the reaction force of the vibrator, when fluids with different acoustic velocity are ejected, are shown in Figure 13. We can see that the effect of acoustic velocity on the reaction force of the vibrator, when it vibrates at low frequency, is also very small.

**Figure 14:**
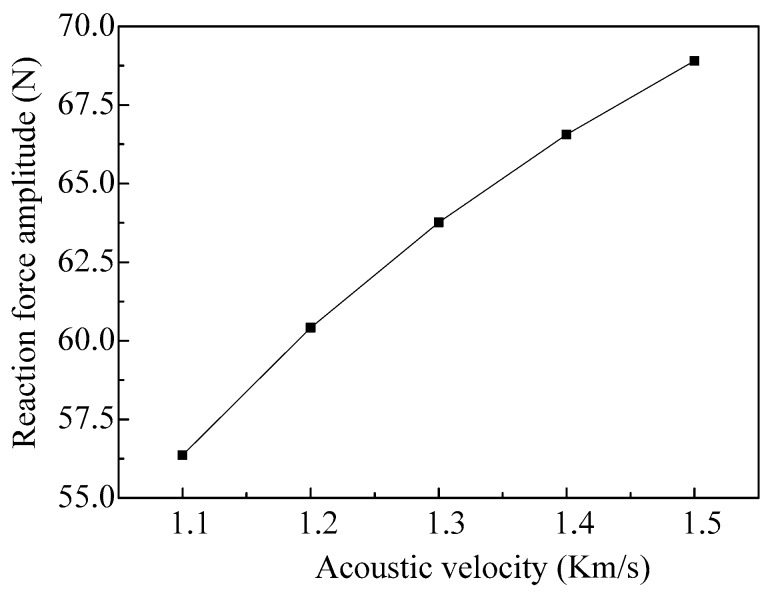
The amplitudes of the reaction force vary with different acoustic velocities of the fluid.

The relationship between the reaction force amplitude of the vibrator working in the fourth-order mode and the acoustic velocity in the fluid is shown in Figure 14. As we can see, with the increase of the acoustic velocity, the amplitude of the reaction force increases gradually, and the changes of the amplitude decreases.

**Figure 15:**
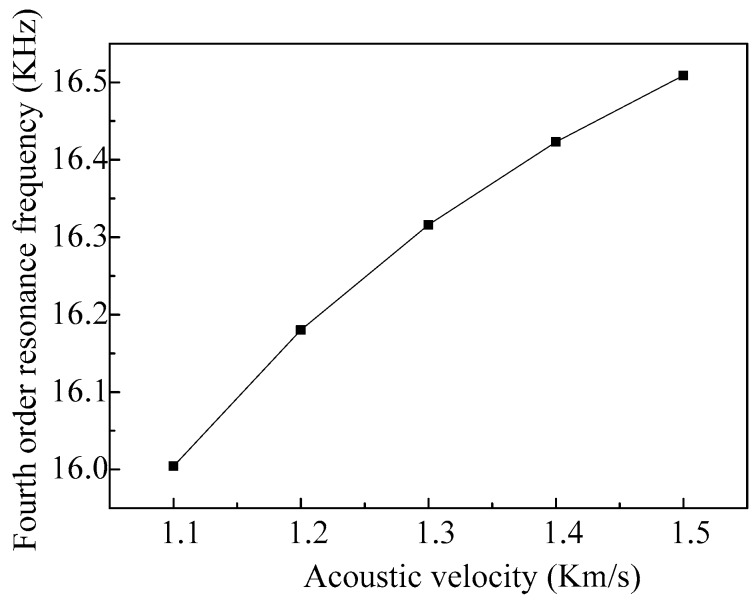
The fourth-order resonance frequencies of the vibrator varies with different fluid densities.

When the vibrator vibrates in the fourth-order mode, the influences of the fluid acoustic velocity on the resonant frequency of the vibrator are shown in Figure 15. We can see that the resonance frequency of the vibrator increases gradually with the increase of the acoustic velocity of the fluid, and the changes of the frequency decrease. Therefore, the operating frequency of the micro-jet increases along with the increase of the acoustic velocity of the fluid.

### 4.3. Influences of the Shell Material

**Figure 16:**
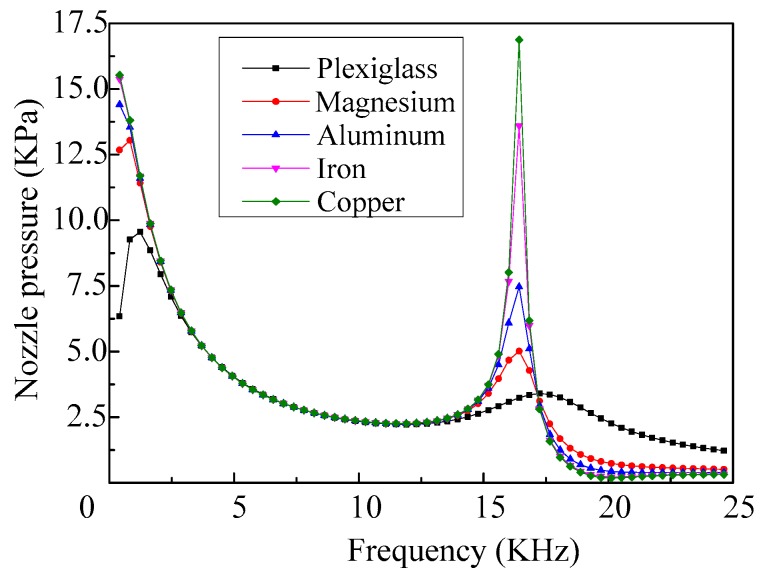
The frequency response curves of the nozzle pressure of the micro-jet (shell with different materials).

When selecting different shell materials and the density and acoustic velocity of the fluid are set as 1000 kg/m^3^ and 1400 m/s, respectively, the frequency response curves of the nozzle pressure of the micro-jet are shown in Figure 16. As we can see, the impedances of the shell materials have little influence on the first-order and fourth-order resonant frequencies of the vibrator, whereas, with the changes of the impedance of the shell material, the changes of the peak value of the nozzle pressure are relatively large.

**Figure 17:**
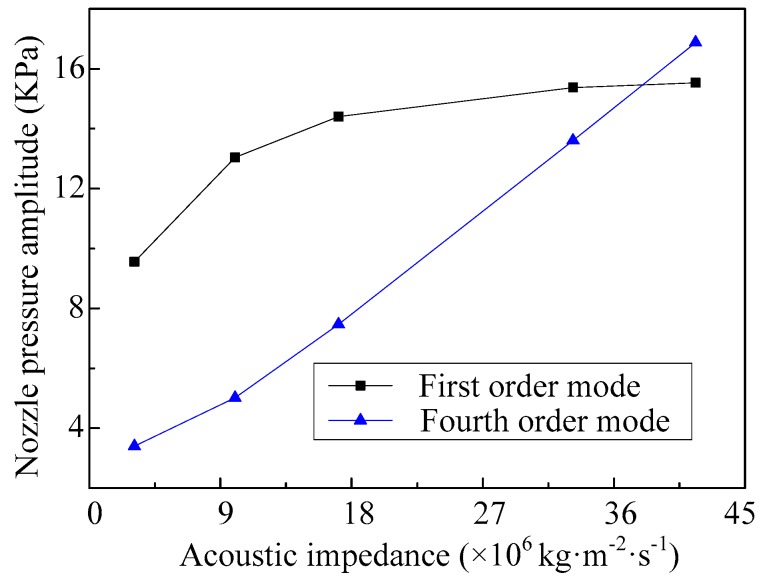
The amplitudes of the nozzle pressure vary with different acoustic impedance of the shell.

The relationship curves between the material impedance of the shell and the nozzle pressure, when the vibrator works in the first-order mode and fourth-order mode, are shown in Figure 17. It can be seen that when the acoustic impedance of the shell material is less than 36 × 10^6^ kg·m^−2^·s^−1^, the nozzle pressure amplitudes of the micro-jet when working in the first-order mode are greater than that of working in the fourth-order mode. With the increase of the acoustic impedance of the shell, the nozzle pressure amplitude increases gradually and the change tends to be stable when the micro-jet is working in the first-order mode. The nozzle pressure amplitude of the micro-jet, working in the fourth-order mode, also increases along with the increase of the acoustic impedance of the shell; however, the change tends to be enhanced.

**Figure 18:**
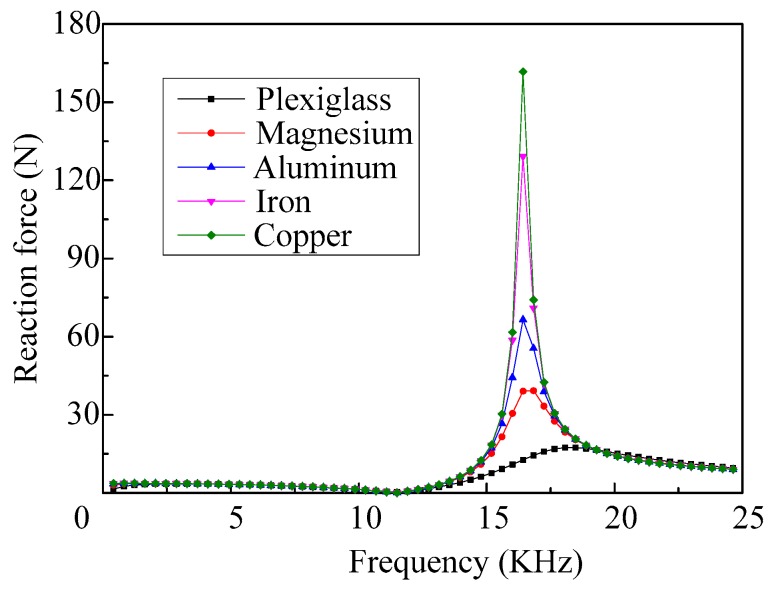
The frequency response curves of the reaction force of the vibrator when selecting shells with different acoustic impedance.

When the shell materials with different acoustic impedances are selected, the response curves of the reaction force of the vibrator are shown in Figure 18. We can see that when the micro-jet works at a low frequency that the reaction force of the vibrator is little affected by the impedance of the shell material. However, the impedance of the shell material has a great influence on the reaction force of the vibrator, when the vibrator works in the fourth-order mode.

**Figure 19:**
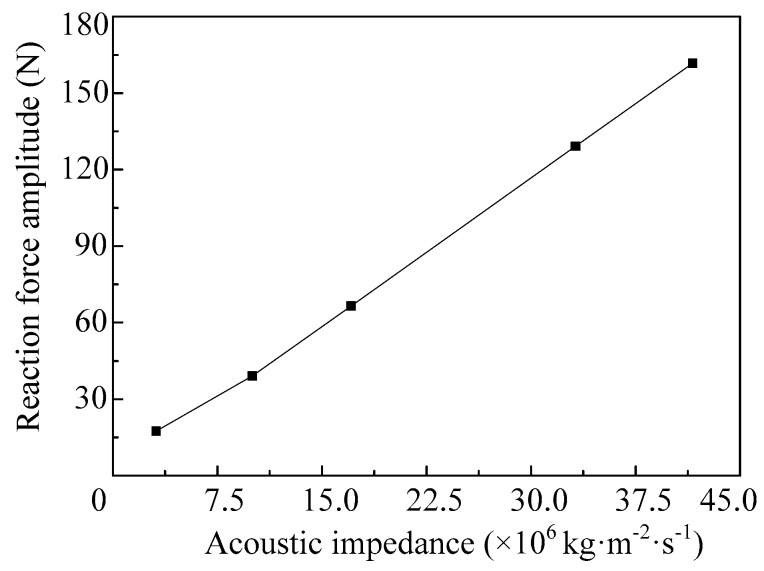
The amplitudes of the reaction force vary with different acoustic impedance of the shell.

The influences of the impedance of the shell material on the reaction force amplitude of the vibrator, when the vibrator works in the fourth-order mode, are shown in Figure 19. We can see that the reaction force amplitude of the vibrator increases near linearly with the increase of the acoustic impedance of the shell.

## 5. Conclusions

When fluid with relatively low density and low acoustic velocity is ejected, the shell material with high acoustic impedance should be selected and the pulse voltage excitation should be enhanced, so as to ensure the strength of the ejection.

In order to guarantee the required restraint stiffness and stability of the vibrator, when fluid with relatively low density and high acoustic velocity is ejected, the shell material with small acoustic impedance is recommended to reduce the reaction force amplitude of the vibrator.

Ejecting fluid with relatively small density and high acoustic velocity, when there are a variety of fluids that can be selected, can increase the working frequency of the micro-jet, which means the working efficiency of the micro-jet can be improved. In addition, the influences of the acoustic impedance of the shell on the operating frequency of the micro-jet can be neglected.

## Figures and Tables

**Table 1:** Acoustic impedance of different materials (×10^6^ Kg·m^−2^·s^−1^).

Material	Plexiglass	Magnesium	Aluminum	Iron	Copper
Impedance	3.1	10.0	17.1	33.2	41.6
